# Genetic diversity and distinction of *Enterococcus faecium* and *Enterococcus lactis* in traditional Montenegrin brine cheeses and salamis

**DOI:** 10.3389/fmicb.2024.1473938

**Published:** 2024-12-11

**Authors:** Beatriz Daza Prieto, Nadja Raicevic, Aleksandra Martinovic, Johann Ladstätter, Ivana Zuber Bogdanovic, Anika Schorpp, Anna Stoeger, Robert L. Mach, Werner Ruppitsch, Adriana Cabal

**Affiliations:** ^1^Institute of Medical Microbiology and Hygiene, Austrian Agency for Health and Food Safety, Vienna, Austria; ^2^Centre of Excellence for Digitalisation of Microbial Food Safety Risk Assessment and Quality Parameters for Accurate Food Authenticity Certification, University of Dona Gorica, Podgorica, Montenegro; ^3^Institute for Animal Nutrition and Feed, Austrian Agency for Health and Food Safety, Linz, Austria; ^4^Institute of Chemical, Environmental and Bioscience Engineering, Research Area of Biochemical Technology, Technical University Vienna, Vienna, Austria; ^5^Department of Biotechnology, University of Natural Resources and Life Sciences, Vienna, Austria

**Keywords:** whole genome sequencing, *Enterococcus faecium*, *Enterococcus lactis*, antimicrobial resistance genes, virulence genes, food safety, traditional food

## Abstract

**Introduction:**

*Enterococcus faecium* is a widespread acid-lactic bacterium found in the environment, humans, and animal microbiota, and it also plays a role in the production of traditional food. However, the worldwide emergence of multidrug-resistant *E. faecium* strains represents a major public health threat and is the primary reason that the genus *Enterococcus* is not recommended for the Qualified Presumption of Safety (QPS) list of the European Food Safety Authority (EFSA), raising concerns about its presence in food products.

**Methods:**

In this study, 39 *E. faecium* and 5 *E. lactis* isolates were obtained from artisanal brine cheeses and dry sausages, sourced from 21 different Montenegrin producers. The isolates were collected following the ISO 15214:1998 international method and processed for whole-genome sequencing (WGS).

**Results:**

Genome analysis based on core genome multilocus sequence type (cgMLST) revealed a high diversity among isolates. Furthermore, the isolates carried antimicrobial resistance genes; the virulence genes *acm*, *sgrA*, and *ecbA*; the bacteriocin genes *Enterolysin A*, *Enterocin A, Enterocin P, Duracin Q, Enterocin B, Bacteriocin 31, Enterocin EJ97, Sactipeptides*, and *Enterocin SEK4*; the secondary metabolite genes *T3PKS*, cyclic lactone autoinducer, RiPP-like, and *NRPS* and a maximum of eight plasmids.

**Conclusion:**

This study highlights the need for careful monitoring of *E. faecium* and *E. lactis* strains in food to ensure they do not pose any potential risks to consumer safety.

## Introduction

*Enterococcus* sp. are Gram-positive and facultative anaerobe lactic acid bacteria (LAB). Within this genus, 63 of the 87 identified species are validly published according to the German Collection of Microorganisms and Cell Culture GmbH (DSMZ; https://lpsn.dsmz.de/search?word=Enterococcus). Among them, *Enterococcus faecium* (*E. faecium*) represents one of the most ubiquitous species, as it is a commensal microorganism of the gastrointestinal tract of humans and animals, but it can also be found in environmental niches, such as soil, water, plants, or food (Delpech et al., 2012). In the latter, *E. faecium* is one of the most prevalent bacteria in traditionally manufactured cheeses (Serio et al., 2007), meat (Lauková et al., 2020), fermented vegetables, and fermented milk (Ben Belgacem et al., 2009), where it provides several beneficial effects, acting either as a probiotic (Foulquié Moreno et al., 2006; Jahansepas et al., 2019), or contributing to specific aroma, flavor, and taste (Delpech et al., 2012) during the fermentation process through the generation of flavor compounds (Abeijón et al., 2006). In several countries, the use of non-starter LAB is indispensable for producing traditional cheese and meat products (Delpech et al., 2012; Abeijón et al., 2006; Yerlikaya and Akbulut, 2019; Giraffa, 2003; Tsanasidou et al., 2021), suggesting that *E. faecium* endemic bacterial strains might have specific properties that make them a valuable resource for future food production (Daza Prieto et al., 2023; Woods et al., 2019; Ruppitsch et al., 2021). The UK Advisory Committee on Novel Foods and Processes (ACNFP) previously approved the use of *E. faecium* strain K77D as a starter culture in fermented dairy products (Hanchi et al., 2018). Additionally, *E. faecium* is a producer of enterocins, and numerous studies have aimed at their purification and characterization, as well as to determine their potential as a preservative in dairy products. For instance, *E. faecium* RZS C5 produces enterocins with activity against foodborne pathogens in milk and cheese (Hanchi et al., 2018; Huang et al., 2013; Leroy et al., 2003).

Numerous studies have demonstrated the benefits of enterococci as additives in food production (Santos et al., 2015; Fugaban et al., 2021; sim et al., 2018). However, other studies have shown that enterococci also play an important role as contaminants in foods, leading to spoilage (Giraffa, 2002). Additionally, their use has become controversial due to the increasing acquisition of multiple antimicrobial resistances, the most concerning the emergence of vancomycin-resistant *E. faecium* (*VREfm*) clones and their adaptation to the hospital environment (Lee et al., 2019; De Oliveira et al., 2020; Leclercq et al., 1988). The World Health Organization (WHO) classifies *VREfm* as a priority pathogen (Tacconelli et al., 2018) and this is one of the reasons why the genus *Enterococcus* has not been recommended for the Qualified Presumption of Safety (QPS) list of the European Food Safety Authority (EFSA; Koutsoumanis et al., 2023) neither has obtained the “generally recognized as safe” (GRAS) status of food additives that was first described in 1958 by the United States Food and Drug Administration (FDA) in the Food Additives Amendment of 1958 (1958).

The rapid evolution of NGS technologies, combined with the dual role of *E. faecium* in food microbiology and public health—as beneficial additives or starters in food production, as well as contaminants causing spoilage or carrying antimicrobial resistance genes (ARGs) or virulence determinants—has resulted in an increasing number of genomic studies that aimed to distinguish between food-grade and pathogenic strains (Martín-Platero et al., 2009; Montealegre et al., 2016).

As a result, three different *E. faecium* “lineages” or “clades” have been described: clade A1, which includes hospital-associated strains; clade A2, which includes animal-associated strains; and clade B, which includes commensal/community-associated strains (Montealegre et al., 2016). The latter was recently reassigned to *E. lactis* in a genome-based study (Belloso Daza et al., 2021). Since then, several groups focused their studies on this re-classification of *E. faecium* clade B (Belloso Daza et al., 2022). The best example of *E. faecium* strains with different evolutionary trajectories is *E. faecium* clonal complex 17 (CC17; clade A1 hospital-associated *E. faecium*) and *E. faecium* clonal complex 94 (CC94; clade B community/commensal *E. faecium*; Peng et al., 2022). Additionally, several studies have reported that *E. faecium* clade A1 strains differ up to 5% from *E. faecium* clade B (now re-classified as *E. lactis*) strains (Galloway-Peña et al., 2012; Wei et al., 2024). CC17 *E. faecium* is the most dominant CC in hospital environments and is the main responsible for healthcare-associated infections worldwide due to its ability to acquire and disseminate ARGs (Lee et al., 2019; De Oliveira et al., 2020). On the other hand, community-associated *E. faecium* are commensal strains that are part of the human microbiome participating in the metabolism of nutrients and synthesis of vitamins (Krawczyk et al., 2021). Regarding antimicrobial resistance (AMR), *E. faecium* and *E. lactis* are generally intrinsically resistant to aminoglycosides, macrolides, and pleuromutilin antibiotics. As a result, these antibiotics are not commonly used to treat infections. Instead, vancomycin, linezolid, daptomycin, tigecycline, quinupristin/dalfopristin, and ampicillin are preferred. However, the usage of vancomycin and ampicillin has decreased, primarily due to the growing resistance to these antibiotics highlighting the need for new therapeutic options (Arias and Murray, 2012). Community-associated *E. faecium* and *E. lactis* strains are more often susceptible to the antibiotics typically used to treat *E. faecium* infections compared to clinical strains. However, some vancomycin-resistant strains have also been described among the community-associated isolates (D’Agata et al., 2001; Schwalbe et al., 1999). A crucial aspect to consider an *E. faecium* strain as “safe” is the absence of the ARGs *vanA*/*vanB*, the virulence genes (VGs) *hylEfm* and *esp.,* and the insertion sequence *IS16*, as required by the European Food Safety Authority (EFSA; EFSA Panel on Additives and Products or Substances used in Animal Feed (FEEDAP) et al., 2018). While clinically associated *E. faecium* strains typically carry the above-mentioned genetic markers, these markers are generally absent in community-associated *E. faecium* strains.

While *VREfm* is responsible for numerous outbreaks worldwide (Zhou et al., 2018; Gorrie et al., 2019), *E. lactis* is a commensal in the gastrointestinal tract of humans and animals and some strains of this species are used in the clinic for gastrointestinal disorders such as acute diarrhea (Holzapfel et al., 2018). The present study aimed to characterize *E. faecium* and *E. lactis* strains present in traditionally produced Montenegrin food products using whole-genome sequencing and to assess their safety status via comparison to hospital-associated strains and investigation of ARGs, VGs, bacteriocins, secondary metabolites, plasmids, and chromosomal point mutations.

## Materials and methods

### Origin and cultivation of isolates

In this study, 25 white brine cheeses and 13 beef and pork dry sausages were collected between 2016 and 2022 from producers located in different municipalities/cities in Montenegro (Supplementary Figure 1 and Supplementary Table 1).

Cheese and salami samples, sourced from diverse origins and collected over different time periods as part of two distinct projects (detailed in the funding information), were processed using the ISO 15214:1998 method (International Organization for Standardization, 1998). Briefly, samples were homogenized using a stomacher and subjected to decimal dilutions (10^−1^–10^−6^) in buffered peptone water (Thermo Scientific/Vienna/Austria). From each dilution, 0.1 ml aliquots were inoculated on De Man, Rogosa, and Sharpe agar (MRS) and M17 (Thermo Scientific, Vienna, Austria) agar and subsequently incubated under anaerobic conditions at 30 and 37°C for 72 and 48 h, respectively. For species identification, up to five single colonies/samples were selected and grown on Columbia Blood Agar with 5% Sheep Blood (COS; BioMérieux, Vienna, Austria) plates at 37°C for 24 h using matrix-assisted laser desorption/ionization time-of-flight mass spectrometry (MALDI-TOF-MS) on a microflex LT/SH (Bruker, Billerica, MA, USA) with the database MBT Compass IVD 4.2. A total of 39 *E. faecium* and 5 *E. lactis* isolates were identified from the 38 food samples and further analyzed.

The isolates from 2019 were designated with the ID INF-X, while those isolated in 2022 were named using the prefix CoE-XX-22.

### DNA extraction and whole-genome sequencing

High-molecular-weight DNA was isolated from overnight cultures grown on COS agar using the MagAttract HMW DNA Kit (Qiagen, Hilden, Germany) following the manufacturer’s instruction for Gram-positive bacteria. DNA purity was quantified using DropSense 16 (Trinean NV/SA, Gentbrugge, Belgium). Genomic libraries were prepared using the Nextera XT DNA library preparation kit (Illumina, San Diego, CA, USA). Paired-end sequencing was performed on a NextSeq2000 instrument (Illumina, San Diego, CA, USA) with a read length of 2 × 150 bp (Illumina, San Diego, CA, USA) and a minimum coverage of 30×.

### Sequence data analysis

Raw reads were *de novo* assembled using SPAdes (version 3.11.1; Bankevich et al., 2012). *Contigs* were filtered for a minimum coverage of 5 and a minimum length of 200 base pairs using SeqSphere+ software v8.5.1 (Ridom, Münster, Germany). QC parameters of assembled genomes are shown in Supplementary Table 2. Confirmation of species identification was conducted by whole-genome pairwise comparison analysis, 16S rDNA analysis, (https://tygs.dsmz.de, accessed on 25th March 2024), and ANI analysis v3.9.7 (Richter et al., 2016). The Type Strain Genome Server analysis (TYGS) tool from the German Collection of Microorganisms and Cell Cultures GmbH (DSMZ) was used for digital DNA–DNA hybridization (dDDH) using the d4 formula as recommended for draft genomes.

Isolate typing was conducted using SeqSphere+ software v9.0.10 including classical multilocus sequence typing (MLST; Homan et al., 2002) and core genome (cg)MLST (Mark de et al., 2015; comprising 1,423 core genome targets). Their genetic relatedness was assessed and for additional genomic comparisons, 31 *E. faecium* and 31 *E. lactis* genome assemblies were downloaded from pubMLST,^1^ from a genome-based study on *E. faecium* performed in 2021 (Belloso Daza et al., 2021) and GenBank. A Minimum Spanning Tree (MST) with all isolates was generated to visualize clusters by applying a cluster threshold set to ≤10 allelic differences. Additionally, a neighbor-joining tree of the core genome alignment of all isolates was visualized and annotated with iTOL (Letunic and Bork, 2024).

NCBI AMRFinder+ v3.11.2. (Letunic and Bork, 2024) and tools from the Center for Genomic Epidemiology (https://www.genomicepidemiology.org/; accessed on 14 June 2024) were used to detect ARGs. All matches for ARGs fulfilling the recent EFSA guidelines of at least 80% identity and 70% query coverage (EFSA, 2024) were reported. Antimicrobial resistance to antibiotics (amikacin, kanamycin, streptomycin, ampicillin, ciprofloxacin, erythromycin, and tetracycline) was tested using E-test (BioMérieux, Vienna, Austria). The virulence factor database (VFDB) was used to detect VGs (Liu et al., 2022). Thresholds were set for the target scanning procedure to ≥85% identity with the reference sequence and ≥99% with the aligned reference sequence. The CGE Mobile Element Finder v1.0.5. was used with the <90% identity and >95% alignment method to detect mobile genetic elements (MGEs; Johansson et al., 2021). Chromosome and Plasmid Finder v1.0. through MOB-suite v3.1.4. available in SeqSphere+ v8.5.1. was used with the mash neighbor distance >0.06 to detect plasmids (Robertson and Nash, 2018). BAGEL4 (van Heel et al., 2018) was used to detect bacteriocins and antiSMASH 7.0 (Blin et al., 2023) to detect secondary metabolite “biosynthetic gene clusters.”

## Results

### Whole-genome sequence-based species identification and subtyping

Digital DNA–DNA hybridization using formula d4 (cutoff >70%; Supplementary Figure 2 and Supplementary Table 3) and ANI analysis (cutoff >95% identity; Supplementary Tables 4.1, 4.2) identified 39 isolates as *E. faecium* and 5 isolates as *E. lactis*.

**Figure 1:**
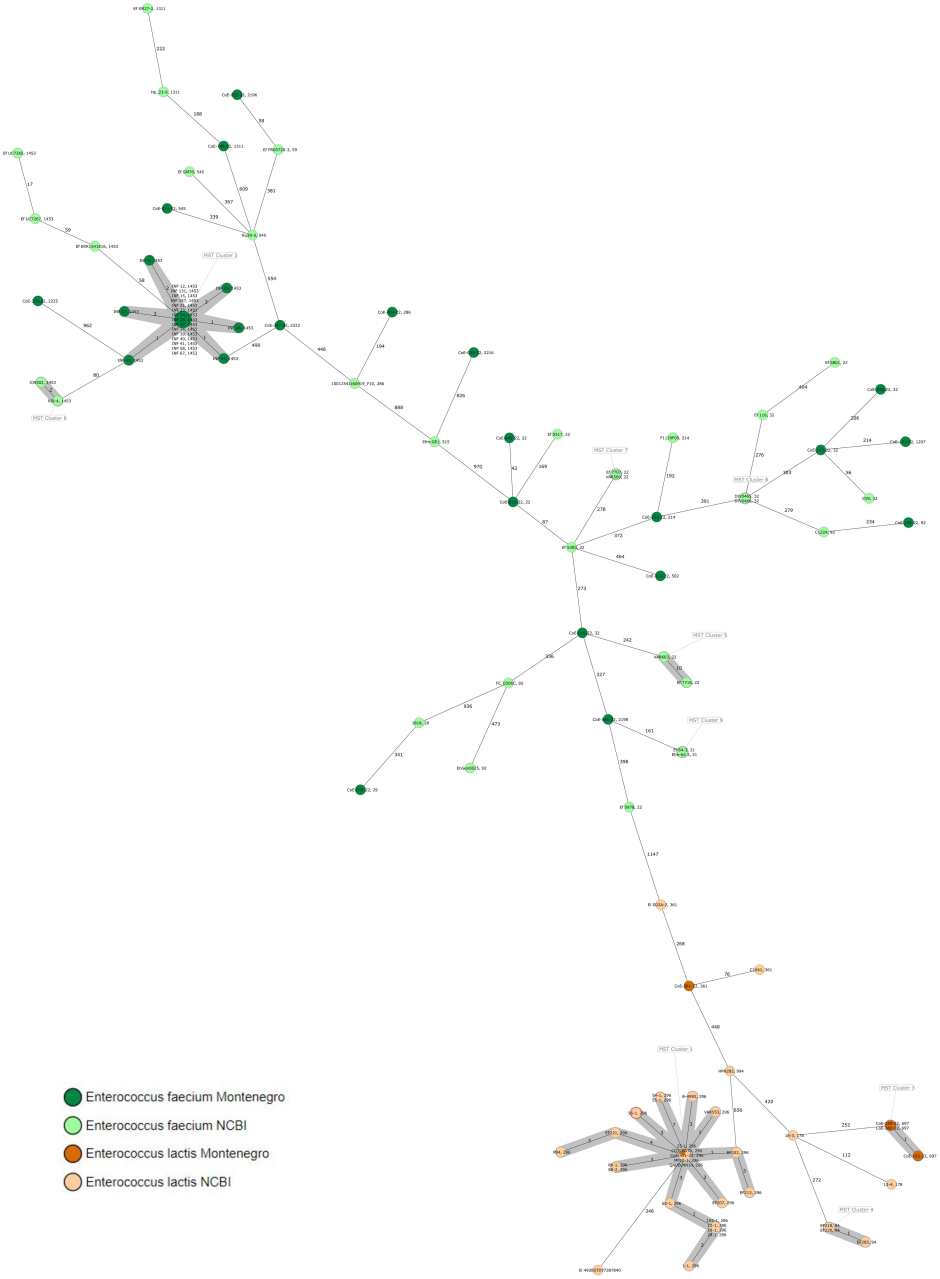
Minimum spanning tree (MST) based on cgMLST analysis of our *E. faecium* (*n* = 39) and *E. lactis* (*n* = 5) isolates and the closest publicly available isolates. Numbers on connection lines represent allelic differences between isolates. Isolates are colored by species (dark gree*n* = *E. faecium* isolates from our study, light green: *E. faecium* isolates from the study performed by Belloso Daza et al. (2021), GenBank and PubMLST, dark orange = *E. lactis* isolates from our study and light orange: *E. lactis* isolates from the study performed by Belloso Daza et al. (2021), GenBank and PubMLST). The threshold for cluster identification is set at ≤10 allelic differences. Data are shown in every isolate: isolate ID and sequence type (ST).

MLST- and cgMLST-based characterization of the 39 *E. faecium* isolates revealed a high diversity. *E. faecium* isolates belonged to 16 different sequence types (STs) and 19 different cgMLST complex types (CTs). A total of 21 *E. faecium* isolates were grouped into one cgMLST cluster (cluster 2) and 18 singletons were identified (*n* = 39). Cluster 2 (ST1453/CT2909) isolates were from 18 different cheeses from 11 different producers. All cluster 2 *E. faecium* isolates differed from each other by a maximum of five alleles. A total of 18 *E. faecium* singletons belonged to 15 different ST profiles and 18 different CT profiles differing by 42–1,000 alleles and were obtained from 7 different cheeses and 11 dry sausages from 9 different producers (Figure 1).

The MLST- and cgMLST-based characterization of the five *E. lactis* isolates obtained from five different dry sausages revealed that three isolates (CC94/ST697/CT6825) grouped in the same cluster (cluster 4). One *E. lactis* isolate was a singleton and differed by a minimum of 566 alleles from cluster 4 (Figure 1).

The *E. lactis* isolates CC94/ST296/CT426 (CoE-451-22) clustered in cluster 1 with 22 publicly available *E. lactis* strains from various origins, showing only one to seven allelic differences: 12 isolates obtained from probiotics and one from an unknown source from China, three isolates obtained from probiotics and one isolate from an unknown source from USA, one bovine isolate from Belgium, one isolate obtained from a cow from Russia, one stool isolate from a hospitalized patient from Brazil, one isolate from yogurt from Canada, and one isolate from fermented milk from an unknown country (Supplementary Table 5). All 23 strains showed a maximum allelic difference of 7 to *E. lactis* NCIMB 10415.

Based on cgMLST analysis, *E. lactis* isolates showed a lower percentage of good core targets (maximum 95.7% in comparison to *E. faecium* isolates 99.4%).

### Detection of antimicrobial resistance genes, virulence genes, bacteriocin, secondary metabolites genes, plasmids, and chromosomal point mutations

**Figure 2:**
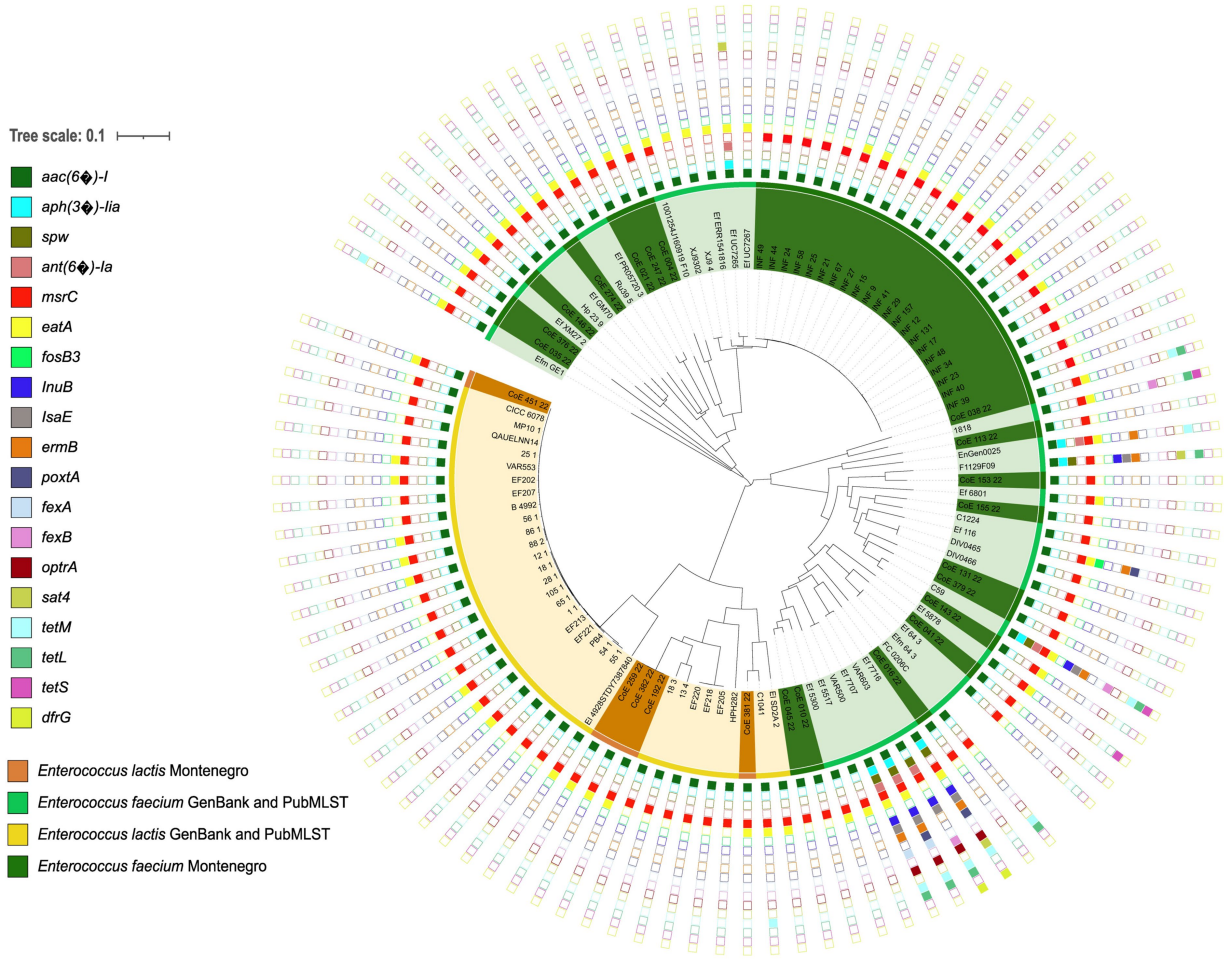
Neighbor-joining phylogenetic tree represents the investigated *E. faecium* (*n* = 70) and *E. lactis* (*n* = 36) based on the core genome alignment of the genomes. Origin and ARGs are displayed for each isolate.

Genome analysis with AMRFinder+ revealed that our *E. faecium* and *E. lactis* isolates carried the intrinsic ARG *aac-(6′)-I* (conferring resistance to aminoglycosides) and *msrC* (conferring resistance to macrolides). Five *E. lactis* isolates from two different producers and 15 *E. faecium* isolates from 12 producers carried the intrinsic ARG *eatA* (conferring resistance to pleuromutilins; Figure 2). Susceptibility testing for erythromycin showed susceptibility in all *E. faecium* and *E. lactis* tested isolates. Susceptibility testing for aminoglycosides (amikacin, kanamycin, and streptomycin) showed antibiotic resistance in all tested *E. faecium* and *E. lactis* isolates. None of the enterococci isolates carried *van* genes. One *E. faecium* singleton (CoE-038-22) isolated from beef sausage possessed the acquired *tetL* and *tetM* ARGs encoded on a plasmid, conferring resistance to tetracycline (Supplementary Table 6). Susceptibility testing of the strain revealed resistance to tetracycline (32 μg/ml). Genome analysis with ResFinder revealed that all *E. faecium* isolates carried a minimum of 4 and a maximum of 17 mutations simultaneously in the class B penicillin-binding protein 5 (*pbp5*) gene (Supplementary Table 7), associated with resistance to ampicillin. Susceptibility testing of a subset of six strains of enterococci showed that all were susceptible to ampicillin (0.19–0.50 μg/ml). *E. lactis* isolates did not carry mutations in *pbp5*. LRE-Finder predicted that all *E. faecium* isolates and *E. lactis* isolates were linezolid susceptible. VFDB showed that 1 *E. lactis* isolate and 13 *E. faecium* isolates (*n* = 14) carried at least 1 VG. Twelve *E. faecium* isolates carried *acm* (encoding a protein responsible for collagen adhesion), two *E. faecium* isolates carried *ecbA* (encoding a protein which binds to the collagen type V), and one *E. lactis* isolate carried *sgrA* (encoding a surface adhesion protein). VirulenceFinder revealed that all enterococci isolates carried the VGs *acm* and *efaAfm* (encoding a protein responsible for cell wall adhesion) with a minimum of 90% identity (Supplementary Table 6). All *E. faecium* isolates and four *E. lactis* isolates (*n* = 43) carried at least one MGE. The most prevalent MGEs were the insertion sequence *ISEf1* and *IS1062*, both found in 23 isolates, followed by the insertion sequences *ISS1N* and *ISEfm,* found in 18 and 14 isolates, respectively (Supplementary Table 6). According to EFSA guidelines for the safety assessment of *E. faecium* strains, none of the isolates carried the VGs *hylEfm* and *esp,* and the insertion sequence *IS*16 (EFSA Panel on Additives and Products or Substances used in Animal Feed (FEEDAP) et al., 2018). MOB-suite database and PlasmidFinder detected 33 Montenegrin *E. faecium* isolates and 5 *E. lactis* isolates that carried at least 1 plasmid (Supplementary Table 6). Six *E. faecium* isolates carried no plasmids. BAGEL4 analysis revealed that 33 isolates carried *Enterolysin A* genes, 8 isolates carried *Enterocin_A* genes, 7 isolates carried *Enterocin_X_chain_alpha* genes, and 5 carried *Enterocin_P* genes. Four isolates carried no genes for bacteriocins (Supplementary Table 8). AntiSMASH 6.0 predicted that all *E. faecium* isolates and *E. lactis* isolates carried type III polyketide synthase (T3PKS) and cyclic lactone autoinducer genes. Thirty-nine out of 44 isolates carried the ribosomally synthesized and post-translationally modified peptides (RiPP-like cluster), and 1 *E. lactis* isolate (CoE-451-22) carried the NRPS cluster (Supplementary Table 8).

## Discussion

Vancomycin-resistant *E. faecium* represents nowadays a significant public health challenge (Koutsoumanis et al., 2023). Conversely, *Enterococcus* species have been described in several countries as essential for the production of artisanal food products, underscoring the importance of correctly distinguishing between beneficial and pathogenic strains to ensure consumer safety. To achieve this, whole-genome sequencing (WGS) is today the method of choice to select safe and beneficial strains in food production (Ruppitsch et al., 2021; EFSA Panel on Additives and Products or Substances used in Animal Feed (FEEDAP) et al., 2023; EFSA Panel on Additives and Products or Substances used in Animal Feed (FEEDAP) et al., 2021; EFSA Panel on Additives and Products or Substances used in Animal Feed (FEEDAP) et al., 2024a; EFSA Panel on Additives and Products or Substances used in Animal Feed (FEEDAP) et al., 2024b; EFSA Panel on Additives and Products or Substances used in Animal Feed (FEEDAP) et al., 2024c).

In our study, WGS revealed a high diversity among *E. faecium* isolates obtained from traditionally produced Montenegrin food, with one large cgMLST cluster (ST1453/CT2909) comprising isolates obtained from brine cheeses in the northern region of Montenegro. ST1453 *E. faecium* isolates were previously reported in traditional brine cheese from Montenegro (Ruppitsch et al., 2020). All ST1453 *E. faecium* isolates carried the intrinsic *aac-(6′)-I* and *msrC* ARGs, while only seven of the isolates possessed the intrinsic *eatA* ARGs. Additionally, five isolates were found to carry the *acm* virulence genes (VGs), and all isolates contained plasmids. To the best of our knowledge, no infections have been linked to ST1453 so far, which suggests that this clone seems to be adapted to artisanal cheese products in Montenegro. This observation is consistent with the review of Giraffa G. (Giraffa, 2003), who reported that enterococci might have a positive influence on cheese due to lipolytic activity, citrate utilization, and production of aromatic volatile compounds. The fact that all our ST1453-positive cheese samples were produced in the same municipality, probably with a similar supply of milk and infrastructures, may explain the colonization with the same *E. faecium* strain.

Regarding the *E. faecium* singletons, ST22, ST32, ST92, and ST296 were the most reported in the literature whereas other detected STs in our study have barely been reported. *E. faecium* ST32 strains have been previously isolated from chicken meat (Leinweber et al., 2018), having an association with vancomycin-resistant genes, which in contrast were absent in our case. *E. faecium* ST22 [which is a single locus variant of *E. faecium* ST32 (Freitas et al., 2020)] was reported by Cabal et al. (2022) with no acquired ARGs nor VGs, which is also in concordance with our findings. As for *E. faecium* ST92, Galloway-Peña et al. (2009) reported its association with vancomycin and ampicillin resistance in clinical isolates and outbreaks in the USA in 1980, which was the first detection of ampicillin resistance in *E. faecium*. In contrast, our *E. faecium* ST92 isolates did not carry any acquired ARGs. However, it carried several mutations in *pbp5* associated with ampicillin resistance. Interestingly, most of the strains in our study, despite having specific mutations in *pbp5* detailed in Supplementary Table 7, which are known to reduce the affinity for ß-lactam antibiotics and confer resistance, were susceptible. Specifically, the point mutations found are nucleotide substitutions that are responsible for increased resistance by decreasing the affinity for ß-lactam antibiotics, leading to ampicillin resistance. On the other hand, other authors such as Rice et al. (2004) reported that the decreased affinity of *pbp5* is not the only factor involved in the expression of resistance to ß-lactams in *E. faecium*. In concordance with our findings, Belloso Daza et al. (2022) reported that some *E. lactis* strains harboring multiple mutations in *pbp5* were still susceptible to ampicillin.

*E. lactis* ST697/CT6825 isolates were obtained from different beef sausages from the same producer in the northeast of Montenegro. This rare ST was reported from a patient isolated in Spain in 2010. Unfortunately, no genome nor a study was available from this isolate for comparison with our isolates. *E. lactis* ST697 strains, lacking VGs, were isolated from sausages from the same producer, therefore we assume that they might be also endemic within the company or producer’s infrastructure. *E. lactis* CC94/ST296/CT426, isolate CoE-451-22 was obtained from sausage and clustered with several isolates from different countries and sources. Due to its relatedness and the similarity of all the other strains within this cluster with *E. lactis* NCIMB 10415, an approved feed additive since 1999 (EFSA Panel on Additives and Products or Substances used in Animal Feed (FEEDAP) et al., 2024c), we hypothesize that *E. lactis* ST296 could have been transferred from the feed to the animals and during slaughter and meat processing to the sausage. In concordance with previous studies, our strain *E. lactis* ST296 carried only intrinsic ARGs (Murray et al., 2020).

The absence of acquired ARGs, including *van* genes, in food-derived enterococci is an important parameter when assessing the safety of these food products for consumers. No vancomycin-resistance-associated ARGs were detected among our strains, which are mostly found in clinical strains, but not in food strains, as reported previously (Jahansepas et al., 2019). Most *E. faecium* and *E. lactis* isolates from our study carried the intrinsic ARGs *aac (6′)-I, eatA,* and *msrC*, which have been described in the literature (Diarra et al., 2010; Shridhar et al., 2022). The presence of intrinsic ARGs alone should not pose any health risk to consumers, as they are resistant to antibiotics that by default are not used in the treatment of clinical *E. faecium* infections. Additionally, some studies reported that macrolide resistance is not solely linked to the presence of the *msrC* gene but depends on specific mutations within the gene (Guido et al., 2001).

Acquired ARGs such as *tetM/tetL*, conferring resistance to tetracycline, were found in one *E. faecium* isolate (ST29) encoded in a mobilizable plasmid, obtained from dry sausage. Although these are acquired ARGs, this is not the first time they have been detected in *E. faecium* isolates obtained from food, specifically from fermented meat products, as reported by Jahan et al. (2013), who reported that the *Enterococcus* sp. isolates included in their study (including 13 *E. faecium*) had 65% resistance to tetracycline. Although *E. faecium* could serve as a reservoir for *tetM/tetL* genes for other bacteria, tetracycline is not a first-line treatment option for humans. None of our strains carried *optrA* ARGs conferring resistance to linezolid. Nevertheless, it is important to note that numerous studies warn about the danger of the presence of the *optrA* gene confirming resistance to linezolid (Xuan et al., 2023). Therefore, monitoring of *optrA* is recommended.

VGs are crucial for the adaptation, pathogenicity, and colonization of bacterial infection (Kim et al., 2016; Fu et al., 2022). None of our isolates carried *hylefm* and *esp* VGs, whose absence is a key aspect according to EFSA guidelines to consider a strain of *E. faecium* as “safe” (EFSA Panel on Additives and Products or Substances used in Animal Feed (FEEDAP), 2012). The VG *acm* and *efaAfm* were present in all *E. faecium* isolates and all *E. lactis* isolates in our study, which is in concordance with other food and probiotics *Enterococcus* studies (Strateva et al., 2016; Yuksekdag et al., 2021). For instance, *acm* has been reported in a fermented dry sausage *E. faecium* isolate from Italy, in the probiotic JDM1 *E. lactis* strain, and in the probiotic SF68 *E. faecium* strain (or *E. faecium* NCIMB 10415, now re-classified as *E. lactis* NCIMB 10415; Belloso Daza et al., 2022; Holzapfel et al., 2018; Fu et al., 2022). The VG *EfaAfm,* one of the most prevalent in *E. faecium* food isolates (Wang et al., 2024), has been obtained from white cheese (Yuksekdag et al., 2021), roe deer and boar meat (Guerrero-Ramos et al., 2016), and in the probiotic *E. faecium* OV3-6 strain (Choeisoongnern et al., 2021). Much less prevalent, *sgrA* was found in one *E. lactis* isolate, and *ecbA* was found in two different *E. faecium* isolates from beef dry sausages (sudzuk). Previous studies reported that the presence of only one of these virulence genes does not indicate pathogenicity; however, the expression and/or combination of these genes could pose a potential risk (Soheili et al., 2014).

All *E. faecium* isolates and *E. lactis* isolates from our study carried plasmids, being the most prevalent rep1 and repUS15. *VREfm* strains were reported harboring *vanA* gene cluster encoded on a rep1 replicon (Yu et al., 2012). While another *VREfm* strain was reported to carry repUS15 plasmid without ARGs (Katsuyama and Ohnishi, 2012). The strain with the acquired *tetM*/*tetL* was encoded on a mobilizable plasmid repUS43, which is in accordance with the results from a recent study (Yu et al., 2012). The presence of plasmids in *E. faecium* strains is considered as a potential risk (Schwalbe et al., 1999; EFSA, 2024), due to the horizontal transfer of ARGs. Therefore, monitoring is essential to mitigate the potential health risks associated with their spread in food.

*E. faecium* isolates obtained from food are reported to produce enterocins, proteins with antimicrobial activity against foodborne pathogens such as *Listeria monocytogenes* (Gontijo et al., 2020; Schittler et al., 2019; Tsigkrimani et al., 2022). Therefore, the application of enterocin-producing *E. faecium* has been described to show potential as a biopreservative against food-borne pathogens (Giraffa, 2003). Our strains carried *Enterolysin A*, *Enterocin A*, *Enterocin P*, *Duracin Q*, *Enterocin B*, *Bacteriocin 31*, *Enterocin EJ97, Sactipeptides,* and *Enterocin SEK4*. *Enterocin A* and *enterocin B* have been reported from *E. faecium* on several occasions (Martín-Platero et al., 2009) and some authors also informed about the presence of *enterocin A*, *enterocin B,* and *enterocin P* in one *E. lactis* strain (Ben Braïek et al., 2018). The most common secondary metabolites in our isolates were T3PKS (polyketides family), cyclic lactone autoinducer, and RiPP-like. The polyketides have been isolated from plants, bacteria, and fungi (Lv et al., 2014) and have been described to have anticancer, anti-cholesterol, and antimicrobial properties (Yu et al., 2012). Specifically, T3PKS in bacteria have significant biological functions such as biosynthesis of some lipidic, natural compounds, and various secondary metabolites, ranging from signaling molecules to bioactive natural products (Katsuyama and Ohnishi, 2012). Furthermore, PKS and RiPP-like cluster genes were detected in two *E. lactis* strains isolated from the rumen of a healthy calf (Korzhenkov et al., 2021), while lactones are used for adding flavors and fragrances to fermented and unfermented dairy products, which seem to have a beneficial use in the making of artisanal cheeses and dry sausages.

In conclusion, all but one investigated *E. faecium* and *E. lactis* isolates carried only intrinsic ARGs and some virulence genes, which alone are not associated with pathogenicity. Some of these isolates may be endemic and could play beneficial roles in traditional cheese and sausage production by enhancing organoleptic qualities and contributing to biopreservation through the production of enterocins, a possibility that warrants further research. However, since all isolates carry plasmids, which is a named risk factor for intentional use according to EFSA recommendations further investigation and ongoing genomic monitoring remain crucial to ensure consumer safety.

## Data Availability

This whole-genome shotgun project has been deposited at DDBJ/ENA/Genbank under the BioProject accession number PRJNA952493. This is the first version of these genomes. The raw sequence reads have been deposited in the Sequence Read Archive (SRA) under the accession numbers SRR24098416 – SRR24098458.

## Footnotes

^1^ *https://pubmlst.org/bigsdb?db=pubmlst_efaecium_isolates&l=1&page=profiles*
