# Supplementary material for: Genetic diversity and distinction of Enterococcus faecium and Enterococcus lactis in traditional Montenegrin brine cheeses and salamis
Source: Front Microbiol. 2024 Dec 11;15:1473938. doi: 10.3389/fmicb.2024.1473938 (PMC11668737; doi:10.3389/fmicb.2024.1473938)
Supplement: Supplementary file 1 [file Data_Sheet_1.docx]

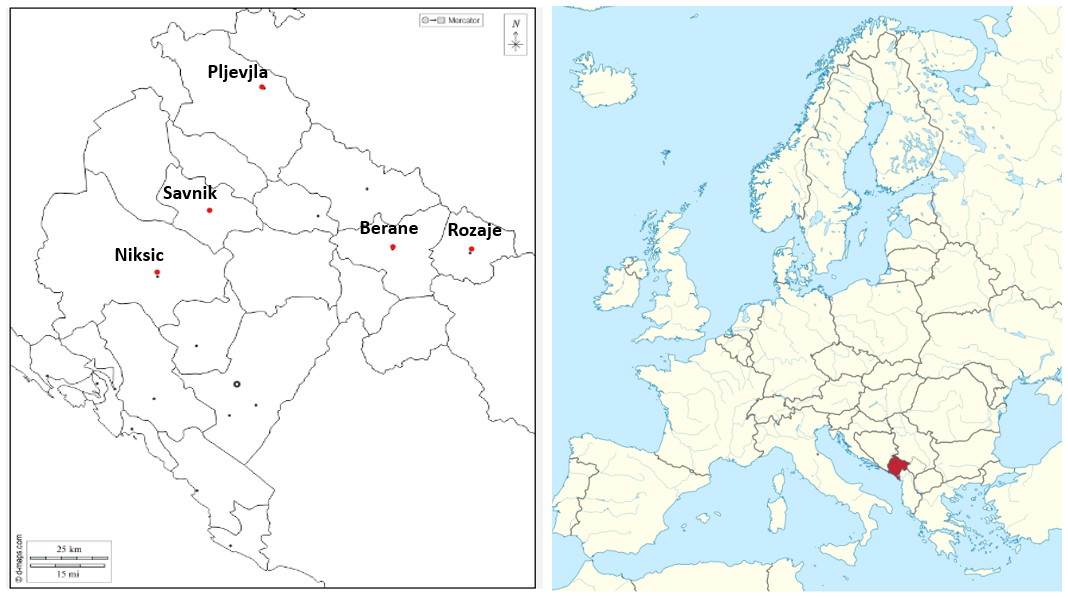


**Supplementary Figure 1.** Montenegro map highlighting in red the municipalities/cities from which food products were collected in this study (left). Map of Europe with Montenegro highlighted in red (right).

**Supplementary Table 1.** Table on isolates ID, species, year of isolation, product, producer and city.

| **Isolate ID** | **Species** | **Year of isolation** | **Product** | **Producer** | **City** | **Reference** |
| --- | --- | --- | --- | --- | --- | --- |
| CoE-451-22 | *E. lactis* | 2016 | Smoked dry pork sausage | A | NikŠić | This study |
| INF9 | *E. faecium* | 2019 | Cheese | B | Pljevlja | Ruppitsch *et al.*  2020 |
| INF12 | *E. faecium* | 2019 | Cheese | B | Pljevlja | Ruppitsch *et al.*  2020 |
| INF15 | *E. faecium* | 2019 | Cheese | C | Pljevlja | This study |
| INF17 | *E. faecium* | 2019 | Cheese | C | Pljevlja | This study |
| INF21 | *E. faecium* | 2019 | Cheese | D | Pljevlja | This study |
| INF23 | *E. faecium* | 2019 | Cheese | D | Pljevlja | This study |
| INF24 | *E. faecium* | 2019 | Cheese | D | Pljevlja | This study |
| INF25 | *E. faecium* | 2019 | Cheese | E | Pljevlja | This study |
| INF27 | *E. faecium* | 2019 | Cheese | E | Pljevlja | This study |
| INF29 | *E. faecium* | 2019 | Cheese | E | Pljevlja | Ruppitsch *et al.*  2020 |
| INF34 | *E. faecium* | 2019 | Cheese | F | Pljevlja | This study |
| INF39 | *E. faecium* | 2019 | Cheese | F | Pljevlja | Ruppitsch *et al.*  2020 |
| INF40 | *E. faecium* | 2019 | Cheese | F | Pljevlja | Ruppitsch *et al.*  2020 |
| INF41 | *E. faecium* | 2019 | Cheese | F | Pljevlja | This study |
| INF44 | *E. faecium* | 2019 | Cheese | G | Pljevlja | This study |
| INF48 | *E. faecium* | 2019 | Cheese | H | Pljevlja | This study |
| INF49 | *E. faecium* | 2019 | Cheese | H | Pljevlja | This study |
| INF58 | *E. faecium* | 2019 | Cheese | I | Pljevlja | This study |
| INF67 | *E. faecium* | 2019 | Cheese | J | Pljevlja | This study |
| INF131 | *E. faecium* | 2019 | Cheese | K | Pljevlja | This study |
| INF157 | *E. faecium* | 2019 | Cheese | L | Pljevlja | This study |
| CoE-004-22 | *E. faecium* | 2022 | Cheese | M | Pljevlja | This study |
| CoE-010-22 | *E. faecium* | 2022 | Beef dry sausage “Giovedi kulen” | N | Rozaje | This study |
| CoE-016-22 | *E. faecium* | 2022 | Beef dry sausage “Giovedi kulen” | N | Rozaje | This study |
| CoE-021-22 | *E. faecium* | 2022 | Cheese | O | Pljevlja | This study |
| CoE-035-22 | *E. faecium* | 2022 | Beef dry sausage “Giovedi sudzuk” | N | Rozaje | This study |
| CoE-038-22 | *E. faecium* | 2022 | Beef dry sausage “Giovedi sudzuk” | N | Rozaje | This study |
| CoE-041-22 | *E. faecium* | 2022 | Beef dry sausage “Giovedi liuta kobasica” | N | Rozaje | This study |
| CoE-045-22 | *E. faecium* | 2022 | Beef dry sausage “Giovedi liuta kobasica” | N | Rozaje | This study |
| CoE-113-22 | *E. faecium* | 2022 | Beef dry sausage “Giovedi kulen” | N | Rozaje | This study |
| CoE-131-22 | *E. faecium* | 2022 | Cheese | P | Pljevlja | This study |
| CoE-143-22 | *E. faecium* | 2022 | Beef dry sausage “Giovedi kulen” | Q | Berane | This study |
| CoE-146-22 | *E. faecium* | 2022 | Cheese | I | Pljevlja | This study |
| CoE-153-22 | *E. faecium* | 2022 | Beef dry sausage “Giovedi kulen” | Q | Berane | This study |
| CoE-155-22 | *E. faecium* | 2022 | Beef dry sausage “Giovedi sudzuk” | Q | Berane | This study |
| CoE-192-22 | *E. lactis* | 2022 | Beef dry sausage “Giovedi kulen” | Q | Berane | This study |
| CoE-247-22 | *E. faecium* | 2022 | Cheese | R | Savnik | This study |
| CoE-259-22 | *E. lactis* | 2022 | Beef dry sausage “Giovedi liuta kobasica” | Q | Berane | This study |
| CoE-274-22 | *E. faecium* | 2022 | Cheese | E | Pljevlja | This study |
| CoE-376-22 | *E. faecium* | 2022 | Cheese | S | Savnik | This study |
| CoE-379-22 | *E. faecium* | 2022 | Beef dry sausage “Giovedi kulen” | Q | Berane | This study |
| CoE-381-22 | *E. lactis* | 2022 | Beef dry sausage “Giovedi liuta kobasica” | Q | Berane | This study |
| CoE-382-22 | *E. lactis* | 2022 | Beef dry sausage “Giovedi liuta kobasica” | Q | Berane | This study |

**Supplementary Table 2.** Information on isolate ID, species, Sequence Type (ST), coverage, contigs, N50 and genome size of the assemblies included in this study.

| **Isolate ID** | **Species** | **Sequence Type (ST)** | **Coverage** | **Contigs** | **N50** | **Genome size** |
| --- | --- | --- | --- | --- | --- | --- |
| CoE-451-22 | *E. lactis* | 296 | 167 | 151 | 57,129 | 2,6 |
| INF9 | *E. faecium* | 1453 | 40 | 287 | 29,445 | 2,7 |
| INF12 | *E. faecium* | 1453 | 41 | 417 | 15,070 | 2,7 |
| INF15 | *E. faecium* | 1453 | 101 | 399 | 18,553 | 2,6 |
| INF17 | *E. faecium* | 1453 | 36 | 586 | 10,615 | 2,6 |
| INF21 | *E. faecium* | 1453 | 96 | 476 | 14,448 | 2,6 |
| INF23 | *E. faecium* | 1453 | 34 | 598 | 9,942 | 2,7 |
| INF24 | *E. faecium* | 1453 | 38 | 476 | 15,155 | 2,7 |
| INF25 | *E. faecium* | 1453 | 30 | 472 | 16,815 | 2,7 |
| INF27 | *E. faecium* | 1453 | 34 | 295 | 39,624 | 2,7 |
| INF29 | *E. faecium* | 1453 | 47 | 623 | 14,675 | 2,8 |
| INF34 | *E. faecium* | 1453 | 41 | 500 | 14,898 | 2,7 |
| INF39 | *E. faecium* | 1453 | 45 | 625 | 9,506 | 2,6 |
| INF40 | *E. faecium* | 1453 | 73 | 289 | 43,961 | 2,7 |
| INF41 | *E. faecium* | 1453 | 151 | 219 | 56,260 | 2,7 |
| INF44 | *E. faecium* | 1453 | 29 | 665 | 8,687 | 2,7 |
| INF48 | *E. faecium* | 1453 | 109 | 375 | 21,640 | 2,7 |
| INF49 | *E. faecium* | 1453 | 38 | 554 | 9,626 | 2,6 |
| INF58 | *E. faecium* | 1453 | 39 | 317 | 24,622 | 2,7 |
| INF67 | *E. faecium* | 1453 | 42 | 543 | 22,272 | 2,7 |
| INF131 | *E. faecium* | 1453 | 62 | 259 | 27,949 | 2,7 |
| INF157 | *E. faecium* | 1453 | 101 | 195 | 54,614 | 2,7 |
| CoE-004-22 | *E. faecium* | 286 | 109 | 266 | 40,270 | 2,7 |
| CoE-010-22 | *E. faecium* | 22 | 173 | 69 | 169,910 | 2,6 |
| CoE-016-22 | *E. faecium* | 32 | 108 | 245 | 43,198 | 2,6 |
| CoE-021-22 | *E. faecium* | 2196 | 111 | 248 | 44,023 | 2,7 |
| CoE-035-22 | *E. faecium* | 2216 | 91 | 239 | 90,911 | 2,5 |
| CoE-038-22 | *E. faecium* | 29 | 126 | 158 | 66,049 | 2,5 |
| CoE-041-22 | *E. faecium* | 2198 | 174 | 194 | 73,489 | 2,5 |
| CoE-045-22 | *E. faecium* | 22 | 164 | 251 | 44,715 | 2,6 |
| CoE-113-22 | *E. faecium* | 502 | 170 | 392 | 146,155 | 2,6 |
| CoE-131-22 | *E. faecium* | 1207 | 62 | 189 | 59,989 | 2,7 |
| CoE-143-22 | *E. faecium* | 32 | 85 | 326 | 35,581 | 2,6 |
| CoE-146-22 | *E. faecium* | 1311 | 96 | 330 | 33,147 | 2,7 |
| CoE-153-22 | *E. faecium* | 214 | 79 | 208 | 50,774 | 2,7 |
| CoE-155-22 | *E. faecium* | 92 | 87 | 178 | 56,790 | 2,7 |
| CoE-192-22 | *E. lactis* | 697 | 104 | 135 | 67,924 | 2,7 |
| CoE-247-22 | *E. faecium* | 2222 | 79 | 403 | 20,000 | 2,6 |
| CoE-259-22 | *E. lactis* | 697 | 125 | 174 | 70,003 | 2,7 |
| CoE-274-22 | *E. faecium* | 545 | 86 | 245 | 44,769 | 2,8 |
| CoE-376-22 | *E. faecium* | 2225 | 79 | 364 | 22,945 | 2,8 |
| CoE-379-22 | *E. faecium* | 32 | 83 | 234 | 34,938 | 2,6 |
| CoE-381-22 | *E. lactis* | 361 | 116 | 308 | 46,260 | 2,6 |
| CoE-382-22 | *E. lactis* | 697 | 90 | 208 | 45,717 | 2,7 |


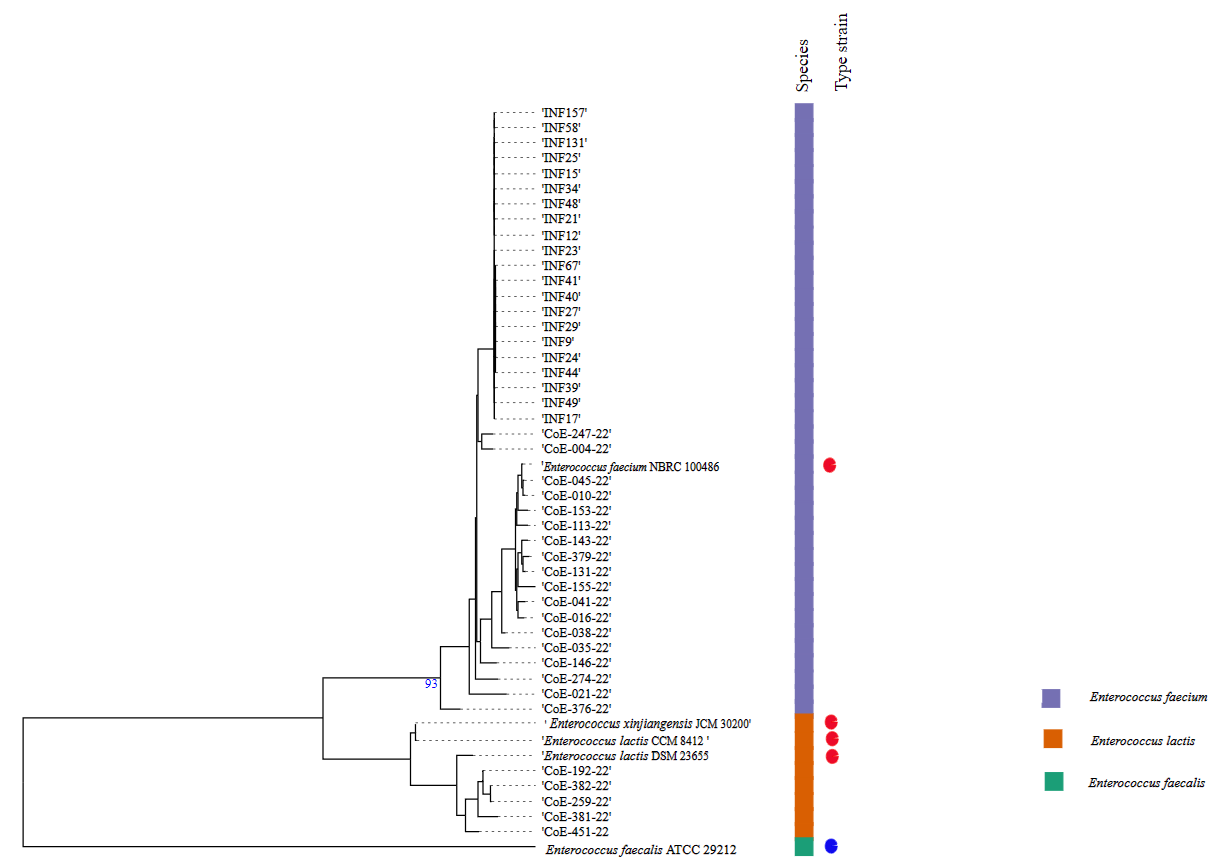


**Supplementary Figure 2.** Comparison analysis tree from TYGS server of 39 Montenegrin Enterococcus faecium isolates and five Enterococcus lactis isolates from brine cheeses and sausages, in addition to type strains Enterococcus faecium NBRC 100486, Enterococcus lactis DSM 23655, Enterococcus lactis CCM 8412 and Enterococcus xinjiangensis JCM 30200 and one type strain of the type species of the genus Enterococcus faecalis ATCC 29212. Thirty-nine Montenegrin Enterococcus faecium strains were identified as Enterococcus faecium being the closest related with TYGS Enterococcus faecium NBRC 100486 strain. Likewise, five Montenegrin Enterococcus lactis strains were identified as Enterococcus lactis being the closest related with TYGS Enterococcus lactis CCM 8412 and Enterococcus xinjiangensis JCM 30200 strains (recently classified as Enterococcus lactis JCM 30200). Red dots indicate type strains; blue dot indicates type species.

| **Supplementary Table 3.** Data on comparison of digital DNA-DNA hybridization (dDDH) of all our 44 Montenegrin enterococci isolates (39 *Enterococcus faecium* and five *Enterococcus lactis*) and the closest type strains available in TYGS server (*Enterococcus faecium* NBRC 100486 and *Enterococcus lactis* JCM 30200). Formula d_4_ is recommended for draft genomes (sum of all identities found in HSPs divided by overall HSP length). HSPs (Heat Shock Proteins). Cut-off >70%. |
| --- |
| \| **Isolate ID** \| **Type Strain** \| **Formula d_4_** \| \| --- \| --- \| --- \| \| CoE-004-22 \| *E. faecium* NBRC 100486 \| 91.4 \| \| CoE-010-22 \| *E. faecium* NBRC 100486 \| 99.5 \| \| CoE-016-22 \| *E. faecium* NBRC 100486 \| 98.5 \| \| CoE-021-22 \| *E. faecium* NBRC 100486 \| 84.9 \| \| CoE-035-22 \| *E. faecium* NBRC 100486 \| 93.5 \| \| CoE-038-22 \| *E. faecium* NBRC 100486 \| 94.3 \| \| CoE-041-22 \| *E. faecium* NBRC 100486 \| 98.1 \| \| CoE-045-22 \| *E. faecium* NBRC 100486 \| 99.5 \| \| CoE-113-22 \| *E. faecium* NBRC 100486 \| 98 \| \| CoE-131-22 \| *E. faecium* NBRC 100486 \| 97.4 \| \| CoE-143-22 \| *E. faecium* NBRC 100486 \| 97 \| \| CoE-146-22 \| *E. faecium* NBRC 100486 \| 91.8 \| \| CoE-153-22 \| *E. faecium* NBRC 100486 \| 98 \| \| CoE-155-22 \| *E. faecium* NBRC 100486 \| 96 \| \| CoE-192-22 \| *E. lactis* JCM 30200 \| 93.2 \| \| CoE-247-22 \| *E. faecium* NBRC 100486 \| 91.3 \| \| CoE-259-22 \| *E. lactis* JCM 30200 \| 93.1 \| \| CoE-274-22 \| *E. faecium* NBRC 100486 \| 89.6 \| \| CoE-376-22 \| *E. faecium* NBRC 100486 \| 91.9 \| \| CoE-379-22 \| *E. faecium* NBRC 100486 \| 96.7 \| \| CoE-381-22 \| *E. lactis* JCM 30200 \| 92 \| \| CoE-382-22 \| *E. lactis* JCM 30200 \| 93 \| \| CoE-451-22 \| *E. lactis* JCM 30200 \| 94.4 \| \| INF9 \| *E. faecium* NBRC 100486 \| 91.6 \| \| INF12 \| *E. faecium* NBRC 100486 \| 91.7 \| \| INF15 \| *E. faecium* NBRC 100486 \| 91.6 \| \| INF17 \| *E. faecium* NBRC 100486 \| 91.8 \| \| INF21 \| *E. faecium* NBRC 100486 \| 91.7 \| \| INF23 \| *E. faecium* NBRC 100486 \| 91.7 \| \| INF24 \| *E. faecium* NBRC 100486 \| 91.7 \| \| INF25 \| *E. faecium* NBRC 100486 \| 91.7 \| \| INF27 \| *E. faecium* NBRC 100486 \| 91.5 \| \| INF29 \| *E. faecium* NBRC 100486 \| 91.6 \| \| INF34 \| *E. faecium* NBRC 100486 \| 91.7 \| \| INF39 \| *E. faecium* NBRC 100486 \| 91.8 \| \| INF40 \| *E. faecium* NBRC 100486 \| 91.5 \| \| INF41 \| *E. faecium* NBRC 100486 \| 91.5 \| \| INF44 \| *E. faecium* NBRC 100486 \| 91.8 \| \| INF48 \| *E. faecium* NBRC 100486 \| 91.7 \| \| INF49 \| *E. faecium* NBRC 100486 \| 91.7 \| \| INF58 \| *E. faecium* NBRC 100486 \| 91.6 \| \| INF67 \| *E. faecium* NBRC 100486 \| 91.5 \| \| INF131 \| *E. faecium* NBRC 100486 \| 91.6 \| \| INF157 \| *E. faecium* NBRC 100486 \| 91.5 \| |

| **Supplementary Table 4.1.** Data on % of ANIb of our 39 E. faecium isolates and type-strain E. faecium NBRC 100486 (cut-off >95%).   \| **Genome** \| **ANIb** \| \| --- \| --- \| \| INF9 \| 98.75 \| \| INF12 \| 98.75 \| \| INF15 \| 98.74 \| \| INF17 \| 98.71 \| \| INF21 \| 98.77 \| \| INF23 \| 98.78 \| \| INF24 \| 98.76 \| \| INF25 \| 98.75 \| \| INF27 \| 98.75 \| \| INF29 \| 98.76 \| \| INF34 \| 98.76 \| \| INF39 \| 98.76 \| \| INF40 \| 98.75 \| \| INF41 \| 98.76 \| \| INF44 \| 98.74 \| \| INF48 \| 98.74 \| \| INF49 \| 98.76 \| \| INF58 \| 98.76 \| \| INF67 \| 98.73 \| \| INF131 \| 98.76 \| \| INF157 \| 98.74 \| \| CoE-004-22 \| 98.70 \| \| CoE-010-22 \| 99.83 \| \| CoE-016-22 \| 99.72 \| \| CoE-021-22 \| 98.09 \| \| CoE-035-22 \| 99.06 \| \| CoE-038-22 \| 99.20 \| \| CoE-041-22 \| 99.68 \| \| CoE-045-22 \| 99.89 \| \| CoE-113-22 \| 99.62 \| \| CoE-131-22 \| 99.62 \| \| CoE-143-22 \| 99.54 \| \| CoE-146-22 \| 98.80 \| \| CoE-153-22 \| 99.69 \| \| CoE-155-22 \| 99.40 \| \| CoE-247-22 \| 98.55 \| \| CoE-274-22 \| 98.62 \| \| CoE-376-22 \| 98.74 \| \| CoE-379-22 \| 99.52 \| | | | | |
| --- | --- | --- | --- | --- | --- | --- | --- | --- | --- | --- | --- | --- | --- | --- | --- | --- | --- | --- | --- | --- | --- | --- | --- | --- | --- | --- | --- | --- | --- | --- | --- | --- | --- | --- | --- | --- | --- | --- | --- | --- | --- | --- | --- | --- | --- | --- | --- | --- | --- | --- | --- | --- | --- | --- | --- | --- | --- | --- | --- | --- | --- | --- | --- | --- | --- | --- | --- | --- | --- | --- | --- | --- | --- | --- | --- | --- | --- | --- | --- | --- | --- | --- | --- | --- |
|  | | | | |
|  | | | | |
|  | | | | |
| **Supplementary Table 4.2.** Data on % of ANIb of our 5 E. lactis isolates and type-strain E. lactis JCM 30200. E. lactis CCM 8412/DSM 23655 and E. faecium NBRC 100486 (cut-off >95%). Data shown all vs all.   \|  \| **CoE-451-22** \| **CoE-382-22** \| **CoE-381-22** \| **CoE-259-22** \| **CoE-192-22** \| ***E. lactis* JCM30200** \| ***E. lactis* CCM8412** \| ***E. faecium* NBRC100486** \| \| --- \| --- \| --- \| --- \| --- \| --- \| --- \| --- \| --- \| \| ***E. lactis* CoE-451-22** \| * \| 99.10 \| 99.14 \| 99.11 \| 99.10 \| 99.16 \| 98.71 \| 94.51 \| \| ***E. lactis* CoE-382-22** \| 98.81 \| * \| 99.16 \| 99.98 \| 99.98 \| 98.66 \| 98.22 \| 94.25 \| \| ***E. lactis* CoE-381-22** \| 98.77 \| 99.14 \| * \| 99.12 \| 99.18 \| 98.43 \| 98.04 \| 94.46 \| \| ***E. lactis* CoE-259-22** \| 98.88 \| 99.95 \| 99.19 \| * \| 99.95 \| 98.69 \| 98.25 \| 94.24 \| \| ***E. lactis* CoE-192-22** \| 98.95 \| 100.00 \| 99.20 \| 99.99 \| * \| 98.83 \| 98.31 \| 94.34 \| \| ***E. lactis* JCM30200** \| 99.19 \| 98.93 \| 98.88 \| 98.95 \| 98.93 \| * \| 98.45 \| 94.46 \| \| ***E. lactis* CCM8412** \| 98.37 \| 98.08 \| 97.95 \| 98.09 \| 98.06 \| 98.17 \| * \| 93.85 \| \| ***E. faecium* NBRC100486** \| 94.60 \| 94.64 \| 94.85 \| 94.65 \| 94.64 \| 94.45 \| 94.35 \| * \| | | | | |
|  | | | | |
|  |  |  |  |  |
| **Supplementary Table 5.** Information on isolate ID, accession number, source, country and ST/CT of Enterococcus lactis ST296 retrieved from pubMLST included in our study. Allelic differences: from isolate E. lactis NCIMB 10415.   \| **Isolate ID** \| **Accession number** \| **Source** \| **Country** \| **ST/CT** \| **Allelic differences** \| \| --- \| --- \| --- \| --- \| --- \| --- \| \| VAR553 \| SRR12858144 \| Animal bovine \| Belgium \| 296/426 \| 1 \| \| 88-2 \| GCF_018397315.1 \| Probiotic product \| China \| 296/426 \| 3 \| \| 86-1 \| GCA_018397375.1 \| Probiotic product \| China \| 296/426 \| 3 \| \| 56-1 \| GCA_018397455.1 \| Probiotic product \| China \| 296/426 \| 3 \| \| EF202 \| GCA_019662465.1 \| Unknown \| USA \| 296/426 \| 1 \| \| EF213 \| GCA_019662245.1 \| Probiotic product \| USA \| 296/426 \| 4 \| \| 1-1 \| GCA_018397815.1 \| Probiotic product \| China \| 296/426 \| 6 \| \| 105-1 \| GCA_018397225.1 \| Probiotic product \| China \| 296/426 \| 4 \| \| 12-1 \| GCA_018397745.1 \| Probiotic product \| China \| 296/426 \| 4 \| \| 18-1 \| GCA_018397675.1 \| Probiotic product \| China \| 296/426 \| 4 \| \| 25-1 \| GCA_018397695.1 \| Probiotic product \| China \| 296/426 \| 0 \| \| 28-1 \| GCA_018397655.1 \| Probiotic product \| China \| 296/426 \| 4 \| \| 55-1 \| GCA_018397485.1 \| Probiotic product \| China \| 296/426 \| 7 \| \| 65-1 \| GCA_018397515.1 \| Probiotic product \| China \| 296/426 \| 3 \| \| B-4992 \| GCA_018069825.1 \| Bos taurus (cow) \| Russia \| 296/426 \| 3 \| \| EF207 \| GCA_019662385.1 \| Probiotic product \| USA \| 296/426 \| 2 \| \| EF221 \| GCA_019662145.1 \| Probiotic product \| USA \| 296/426 \| 4 \| \| MP10-1 \| GCF_017356435.1 \| Spheniscus magellanicus (Magellanic penguin) \| Brazil \| 296/426 \| 0 \| \| PB4 \| GCA_022586915.1 \| Yogurt \| Canada \| 296/426 \| 7 \| \| QAUELNN14 \| GCA_022509645.1 \| Fermented milk product \| Unknown \| 296/426 \| 0 \| \| CICC 6078 \| WOTP00000000.1 \| Unknown \| China \| 296/426 \| 0 \| |  |  |  |  |

**Supplementary Table 6.** Data on ARG (80% identity and 70% query coverage), VGs, MGEs and plasmids.

| **Strain ID** | **Species** | **Antimicrobial Resistance Genes** | **Virulence Genes** | **MGEs** | **Plasmid** |
| --- | --- | --- | --- | --- | --- |
| CoE-451-22 | *E. lactis* | *aac(6´)-I, eatA, msrC* | *Acm, efaAfm* | *ISEfa10*, *ISLgar5*, *ISEnfa4* | Rep29 |
| INF9 | *E. faecium* | *aac(6´)-I, eatA, msrC* | *Acm, efaAfm* | *IS1062*, *ISEf1*, *ISS1N* | Rep1, repUS15 |
| INF12 | *E. faecium* | *aac(6´)-I, eatA, msrC* | *Acm, efaAfm* | ISEfm2, IS1062, ISS1N, ISEf1 | Rep1 |
| INF15 | *E. faecium* | *aac(6´)-I, eatA, msrC* | *Acm, efaAfm* | *ISEfm2*, *IS1062*, *ISEf1* | Rep1, repUS15 |
| INF17 | *E. faecium* | *aac(6´)-I, eatA, msrC* | *Acm, efaAfm* | *IS1062*, *ISEf1* | Rep1, repUS15 |
| INF21 | *E. faecium* | *aac(6´)-I, eatA, msrC* | *Acm, efaAfm* | *ISEfm2*, *IS1062*, *ISEf1* | Rep1, repUS15 |
| INF23 | *E. faecium* | *aac(6´)-I, eatA, msrC* | *Acm, efaAfm* | *IS1062*, *ISS1N*, *ISEf1*, *ISEfm1* | Rep1, repUS15 |
| INF24 | *E. faecium* | *aac(6´)-I, eatA, msrC* | *Acm, efaAfm* | *ISEf1, ISEfm1, IS1062, IS481* | Rep1, repUS15 |
| INF25 | *E. faecium* | *aac(6´)-I, eatA, msrC* | *Acm, efaAfm* | *ISEf1, ISEfm1, IS1062, ISS1N* | Rep1, repUS15 |
| INF27 | *E. faecium* | *aac(6´)-I, eatA, msrC* | *Acm, efaAfm* | *ISEf1*, *IS1062* | Rep1, repUS15 |
| INF29 | *E. faecium* | *aac(6´)-I, eatA, msrC* | *Acm, efaAfm* | *ISEf1*, *IS1062* | Rep1 |
| INF34 | *E. faecium* | *aac(6´)-I, eatA, msrC* | *Acm, efaAfm* | *ISEf1* | Rep1, repUS15 |
| INF39 | *E. faecium* | *aac(6´)-I, eatA, msrC* | *Acm, efaAfm* | *ISEfm1, ISEfm2, ISEf1, IS1062, IS1070, ISS1N* | Rep1, repUS15 |
| INF40 | *E. faecium* | *aac(6´)-I, eatA, msrC* | *Acm, efaAfm* | *IS481*, *ISEf1*, *IS1062* | Rep1, repUS15 |
| INF41 | *E. faecium* | *aac(6´)-I, eatA, msrC* | *Acm, efaAfm* | *ISEf1, ISS1N, IS1062* | RepUS15 |
| INF44 | *E. faecium* | *aac(6´)-I, eatA, msrC* | *Acm, efaAfm* | *ISEfm2, ISEfm1, ISS1N, ISEf1, IS1062* | Rep1, repUS15 |
| INF48 | *E. faecium* | *aac(6´)-I, eatA, msrC* | *Acm, efaAfm* | *ISS1N*, *IS1062* | Rep1, repUS15 |
| INF49 | *E. faecium* | *aac(6´)-I, eatA, msrC* | *Acm, efaAfm* | *ISEfm2, ISEf1, ISS1N, IS1062* | Rep1, repUS15 |
| INF58 | *E. faecium* | *aac(6´)-I, eatA, msrC* | *Acm, efaAfm* | *ISEf1*, *IS1062* | Rep1, repUS15 |
| INF67 | *E. faecium* | *aac(6´)-I, eatA, msrC* | *Acm, efaAfm* | *ISEfm1, ISS1N, ISEf1, IS1062* | Rep1 |
| INF131 | *E. faecium* | *aac(6´)-I, eatA, msrC* | *Acm, efaAfm* | *ISEfm1, IS1062, Isf1, ISS1N* | Rep1, repUS15 |
| INF157 | *E. faecium* | *aac(6´)-I, eatA, msrC* | *Acm, efaAfm* | *ISEf1, ISS1N, IS1062* | Rep1, repUS15 |
| CoE-004-22 | *E. faecium* | *aac(6´)-I, eatA, msrC* | *Acm, efaAfm* | *ISEfm2*, *ISEf1*, *ISS1N* | Rep1, rep29, repUS15 |
| CoE-010-22 | *E. faecium* | *aac(6´)-I, eatA, msrC* | *Acm, efaAfm* | *ISEfa10, ISEfm1, ISEfm2* | **-** |
| CoE-016-22 | *E. faecium* | *aac(6´)-I, eatA, msrC* | *Acm, efaAfm* | *ISEfm1*, *ISEfa10* | **-** |
| CoE-021-22 | *E. faecium* | *aac(6´)-I, eatA, msrC* | *Acm, efaAfm* | *ISLgar5*, *ISS1N* | Rep1, repUS15 |
| CoE-035-22 | *E. faecium* | *aac(6´)-I, eatA, msrC* | *Acm, ecba, efaAfm* | *ISSsu5* | Rep14b |
| CoE-038-22 | *E. faecium* | *aac(6´)-I, eatA, msrC, tetL, cat* | *Acm, efaAfm* | *ISS1N* | Rep22, repUS43 |
| CoE-041-22 | *E. faecium* | *aac(6´)-I, eatA, msrC* | *Acm, efaAfm* | *ISEfa10*, *ISEfm1* | **-** |
| CoE-045-22 | *E. faecium* | *aac(6´)-I, eatA, msrC* | *Acm, efaAfm* | *ISEfm2, ISSsu5, ISEfa10, ISEfm1* | **-** |
| CoE-113-22 | *E. faecium* | *aac(6´)-I, eatA, msrC* | *Acm, efaAfm* | *ISEfa10* | **-** |
| CoE-131-22 | *E. faecium* | *aac(6´)-I, eatA, msrC* | *Acm, efaAfm* | *ISLgar5*, *ISEfm1* | RepUS15, rep29, rep1, rep18a |
| CoE-143-22 | *E. faecium* | *aac(6´)-I, eatA, msrC* | *Acm, efaAfm* | *ISEfa10*, *ISEfa11* | Rep1, repUS15 |
| CoE-146-22 | *E. faecium* | *aac(6´)-I, eatA, msrC* | *Acm, efaAfm* | *ISLhe30*, *ISS1N* | Rep1, Rep2, rep17 |
| CoE-153-22 | *E. faecium* | *aac(6´)-I, eatA, msrC* | *Acm, efaAfm* | *ISEf1*, *ISEfa11* | Rep1, repUS15 |
| CoE-155-22 | *E. faecium* | *aac(6´)-I, eatA, msrC* | *Acm, ecba, efaAfm* | *ISEf1, ISEnfa4, ISEfa11, ISLgar5* | Rep1, repUS15 |
| CoE-192-22 | *E. lactis* | *aac(6´)-I, eatA, msrC* | *Acm, efaAfm* | - | Rep1, repUS15 |
| CoE-247-22 | *E. faecium* | *aac(6´)-I, eatA, msrC* | *Acm, efaAfm* | *ISEfm2, ISLhe30, IS1062* | RepUS15 |
| CoE-259-22 | *E. lactis* | *aac(6´)-I, eatA, msrC* | *Acm, efaAfm* | ISEfm1, ISLgar5, ISEfm2 | Rep1, repUS15 |
| CoE-274-22 | *E. faecium* | *aac(6´)-I, eatA, msrC* | *Acm, efaAfm* | *ISLgar5, ISEfm2, IS1062* |  |
|  |  |  |  |  | Rep1, rep29, repUS15 |
|  |  |  |  |  |  |
| CoE-376-22 | *E. faecium* | *aac(6´)-I, eatA, msrC* | *Acm, efaAfm* | *ISEfa5, ISEnfa4, IS1062, ISS1N* | Rep17, rep29, repUS15, repUS52 |
| CoE-379-22 | *E. faecium* | *aac(6´)-I, eatA, msrC* | *Acm, efaAfm* | *ISEfa11*, *ISLgar5* | Rep1, repUS15 |
| CoE-381-22 | *E. lactis* | *aac(6´)-I, eatA, msrC* | *Acm, efaAfm, sgrA* | *ISS1N, ISLgar5* | RepUS15 |
| CoE-382-22 | *E. lactis* | *aac(6´)-I, eatA, msrC* | *Acm, efaAfm* | *ISEfm1, ISLgar5, ISEfm2* | Rep1, repUS15 |

ARG (NCBI AMR+, ResFinder), VGs (VirulenceFinder, VFDB) and plasmids (PlasmidFinder, MOB-suite) of the Montenegrin isolates.

**Supplementary Table 7.** Information on mutation name, nucleotide change and number of E. faecium from our study carrying mutations in pbp5.

| **Mutation** | **Nucleotide change** | **Number of strains carrying the mutation** |
| --- | --- | --- |
| V24A | gta>gca | 39/39 |
| S27G | agt>ggt | 39/39 |
| R34Q | cgg>cag | 39/39 |
| G66E | gga>gaa | 39/39 |
| A68T | gca>aca | 10/39 |
| E85D | gaa>gat | 10/39 |
| E100Q | gag>cag | 38/39 |
| K144Q | aaa>caa | 39/39 |
| T172A | aca>gca | 38/39 |
| L177I | tta>ata | 37/39 |
| D204G | gac>ggc | 10/39 |
| A216S | gca>tcc | 38/39 |
| T324A | aca>gca | 39/39 |
| N496K | aat>aaa | 38/39 |
| A499T | gca>aca | 38/39 |
| E525D | gag>gat | 38/39 |
| P667S | ccc>tcg | 9/39 |

**Supplementary Table 8.** Data on Strain ID, Sequence Type (ST), bacteriocin genes (BAGEL4) and secondary metabolites (AntiSMASH) of our Montenegrin isolates.

| **Strain ID** | **Species** | **ST** | **Bacteriocin genes** | **Secondary metabolite genes** |
| --- | --- | --- | --- | --- |
| CoE-451-22 | *E. lactis* | 296 | *Enterocin EJ97* | Cyclic-lactone-autoinducer, NRPS, T3PKS |
| INF9 | *E. faecium* | 1453 | *Duracin_Q, enterolysin_A* | cyclic-lactone-autoinducer, T3PKS, RiPP-like |
| INF12 | *E. faecium* | 1453 | *Enterolysin_A* | cyclic-lactone-autoinducer, T3PKS, RiPP-like |
| INF15 | *E. faecium* | 1453 | *Enterolysin_A* | cyclic-lactone-autoinducer, T3PKS, RiPP-like |
| INF17 | *E. faecium* | 1453 | *Enterolysin_A* | cyclic-lactone-autoinducer, T3PKS, RiPP-like |
| INF21 | *E. faecium* | 1453 | *Enterolysin_A* | cyclic-lactone-autoinducer, T3PKS, RiPP-like |
| INF23 | *E. faecium* | 1453 | *Enterolysin_A* | cyclic-lactone-autoinducer, T3PKS, RiPP-like |
| INF24 | *E. faecium* | 1453 | *Enterolysin_A* | cyclic-lactone-autoinducer, T3PKS, RiPP-like |
| INF25 | *E. faecium* | 1453 | *Enterolysin_A* | cyclic-lactone-autoinducer, T3PKS, RiPP-like |
| INF27 | *E. faecium* | 1453 | *Enterocin_X_chain_alpha, enterolysin_A* | cyclic-lactone-autoinducer, T3PKS, RiPP-like |
| INF29 | *E. faecium* | 1453 | *Enterolysin_A* | cyclic-lactone-autoinducer, T3PKS, RiPP-like |
| INF34 | *E. faecium* | 1453 | *Enterolysin_A* | cyclic-lactone-autoinducer, T3PKS, RiPP-like |
| INF39 | *E. faecium* | 1453 | *Enterolysin_A* | cyclic-lactone-autoinducer, T3PKS, RiPP-like |
| INF40 | *E. faecium* | 1453 | *Duracin_Q, enterolysin_A* | cyclic-lactone-autoinducer, T3PKS, RiPP-like |
| INF41 | *E. faecium* | 1453 | *Duracin_Q, enterolysin_A* | cyclic-lactone-autoinducer, T3PKS, RiPP-like |
| INF44 | *E. faecium* | 1453 | *Enterolysin_A* | cyclic-lactone-autoinducer, T3PKS, RiPP-like |
| INF48 | *E. faecium* | 1453 | *Enterolysin_A* | cyclic-lactone-autoinducer, T3PKS, RiPP-like |
| INF49 | *E. faecium* | 1453 | *Enterolysin_A* | cyclic-lactone-autoinducer, T3PKS, RiPP-like |
| INF58 | *E. faecium* | 1453 | *Enterocin_X_chain_alpha, enterolysin_A* | cyclic-lactone-autoinducer, T3PKS, RiPP-like |
| INF67 | *E. faecium* | 1453 | *Enterolysin_A* | cyclic-lactone-autoinducer, T3PKS, RiPP-like |
| INF131 | *E. faecium* | 1453 | *Enterocin_A, enterolysin_A* | cyclic-lactone-autoinducer, T3PKS, RiPP-like |
| INF157 | *E. faecium* | 1453 | *Duracin_Q, enterolysin_A* | cyclic-lactone-autoinducer, T3PKS, RiPP-like |
| CoE-004-22 | *E. faecium* | 286 | *Bacteriocin_31, enterolysin_A* | cyclic-lactone-autoinducer, T3PKS, RiPP-like |
| CoE-010-22 | *E. faecium* |  | *Enterocin_A, Enterocin_B* | cyclic-lactone-autoinducer, T3PKS, RiPP-like |
| CoE-016-22 | *E. faecium* | 32 | - | cyclic-lactone-autoinducer, T3PKS, RiPP-like |
| CoE-021-22 | *E. faecium* | 2196 | *Enterocin_P, enterolysin_A, UviB* | cyclic-lactone-autoinducer, T3PKS, RiPP-like |
| CoE-035-22 | *E. faecium* | 2216 | - | Cyclic-lactone-autoinducer, T3PKS |
| CoE-038-22 | *E. faecium* | 29 | - | Cyclic-lactone-autoinducer, T3PKS |
| CoE-041-22 | *E. faecium* | 2198 | *Enterocin_A, enterocin_X_chain_alpha, UviB* | cyclic-lactone-autoinducer, T3PKS, RiPP-like |
| CoE-045-22 | *E. faecium* | 22 | *Enterocin_A, Enterocin_X_chain_alpha, UviB* | cyclic-lactone-autoinducer, T3PKS, RiPP-like |
| CoE-113-22 | *E. faecium* | 502 | *Enterocin_A, Enterocin_B* | Cyclic-lactone-autoinducer, T3PKS |
| CoE-131-22 | *E. faecium* | 1207 | *Enterocin_A, enterocin_X_chain_alpha, enterolysin_A* | cyclic-lactone-autoinducer, T3PKS, RiPP-like |
| CoE-143-22 | *E. faecium* | 32 | *Enterocin_X_chain_alpha, UviB* | cyclic-lactone-autoinducer, T3PKS, RiPP-like |
| CoE-146-22 | *E. faecium* | 1311 | *Enterocin_P, enterolysina_A* | cyclic-lactone-autoinducer, T3PKS, RiPP-like |
| CoE-153-22 | *E. faecium* | 214 | *Enterocin_A, enterocin_X_chain_alpha, enterolysin_A* | cyclic-lactone-autoinducer, T3PKS, RiPP-like |
| CoE-155-22 | *E. faecium* | 92 | *Enterocin_A, enterolysin_A, UviB* | cyclic-lactone-autoinducer, T3PKS, RiPP-like |
| CoE-192-22 | *E. lactis* | 697 | *Enterocin_P, enterolysin_A* | cyclic-lactone-autoinducer, T3PKS, RiPP-like |
| CoE-247-22 | *E. faecium* | 2222 | *Enterolysin_A* | cyclic-lactone-autoinducer, T3PKS, RiPP-like |
| CoE-259-22 | *E. lactis* | 697 | *Enterolysin_A, sactipeptides* | Cyclic-lactone-autoinducer, T3PKS |
| CoE-274-22 | *E. faecium* | 545 | *Enterocin_A, enterolysin_A, bacteriocin_31* | cyclic-lactone-autoinducer, T3PKS, RiPP-like |
| CoE-376-22 | *E. faecium* | 2225 | *Enterolysin_A* | cyclic-lactone-autoinducer, T3PKS, RiPP-like |
| CoE-379-22 | *E. faecium* | 32 | *Enterocin_B* | cyclic-lactone-autoinducer, T3PKS, RiPP-like |
| CoE-381-22 | *E. lactis* | 361 | *Enterocin_SE_K4, enterocin_P* | cyclic-lactone-autoinducer, T3PKS, RiPP-like |
| CoE-382-22 | *E. lactis* | 697 | *Enterocin_P, enterolysin_A* | cyclic-lactone-autoinducer, T3PKS, RiPP-like |
